# Neem oil increases the efficiency of the entomopathogenic fungus *Metarhizium anisopliae* for the control of *Aedes aegypti* (Diptera: Culicidae) larvae

**DOI:** 10.1186/s13071-015-1280-9

**Published:** 2015-12-30

**Authors:** Simone A. Gomes, Adriano R. Paula, Anderson Ribeiro, Catia O. P. Moraes, Jonathan W. A. B. Santos, Carlos P. Silva, Richard I. Samuels

**Affiliations:** Department of Entomology and Plant Pathology, Universidade Estadual do Norte Fluminense Darcy Ribeiro, Campos dos Goytacazes, RJ CEP 28013-602 Brazil; Departamento de Bioquímica, Universidade Federal de Santa Catarina, 88040-900 Florianópolis, Brazil

**Keywords:** Virulence, Phytochemical, Natural insecticide, Fungus, Vector, Dengue, Biological control

## Abstract

**Background:**

Entomopathogenic fungi are potential candidates for use in integrated vector management and many isolates are compatible with synthetic and natural insecticides. Neem oil was tested separately and in combination with the entomopathogenic fungus *Metarhizium anisopliae* against larvae of the dengue vector *Aedes aegypti*. Our aim was to increase the effectiveness of the fungus for the control of larval mosquito populations.

**Methods:**

Commercially available neem oil was used at concentrations ranging from 0.0001 to 1 %. Larval survival rates were monitored over a 7 day period following exposure to neem. The virulence of the fungus *M. anisopliae* was confirmed using five conidial concentrations (1 × 10^5^ to 1 × 10^9^ conidia mL^−1^) and survival monitored over 7 days. Two concentrations of fungal conidia were then tested together with neem (0.001 %). Survival curve comparisons were carried out using the Log-rank test and end-point survival rates were compared using one-way ANOVA.

**Results:**

1 % neem was toxic to *A. aegypti* larvae reducing survival to 18 % with S_50_ of 2 days. Neem had no effect on conidial germination or fungal vegetative growth *in vitro*. Larval survival rates were reduced to 24 % (S_50_ = 3 days) when using 1 × 10^9^ conidia mL^−1^. Using 1 × 10^8^ conidia mL^−1^, 30 % survival (S_50_ = 3 days) was observed. We tested a “sub-lethal” neem concentration (0.001 %) together with these concentrations of conidia. For combinations of neem + fungus, the survival rates were significantly lower than the survival rates seen for fungus alone or for neem alone. Using a combination of 1 × 10^7^ conidia mL^−1^ + neem (0.001 %), the survival rates were 36 %, whereas exposure to the fungus alone resulted in 74 % survival and exposure to neem alone resulted in 78 % survival. When using 1 × 10^8^ conidia mL^−1^, the survival curves were modified, with a combination of the fungus + neem resulting in 12 % survival, whilst the fungus alone at this concentration also significantly reduced survival rates (28 %).

**Conclusions:**

The use of adjuvants is an important strategy for maintaining/increasing fungal virulence and/or shelf-life. The addition of neem to conidial suspensions improved virulence, significantly reducing larval survival times and percentages.

**Electronic supplementary material:**

The online version of this article (doi:10.1186/s13071-015-1280-9) contains supplementary material, which is available to authorized users.

## Background

Dengue fever is one of the most serious viral diseases vectored principally by the mosquito *Aedes aegypti*, affecting 50–100 million people annually. An estimated 500,000 people with severe dengue require hospitalization each year [1]. Sadly, this year’s epidemic in Brazil resulted in a high death rate amongst the older population, 169 deaths occurring so far in the State of São Paulo, with nine out of ten victims over the age of 60 [2].

Currently there is no vaccine available, which means that the only way of reducing these figures is to control the vector. The main strategies for the control of *A. aegypti* are the elimination of breeding sites and chemical insecticide application. However, these strategies are not preventing regular epidemics of dengue fever and new approaches are therefore urgently needed. One of the main problems associated with chemical control is the rapid development of insecticide resistance, which is particularly serious in Brazil where *Aedes aegypti* larvae have been shown to be resistant to the widely used organophosphate temephos [3], whilst adult mosquitoes have also developed resistance to the pyrethroids cypermethrin and deltamethrin [4].

The use of entomopathogenic fungi against insect disease vectors is currently the subject of extensive research [5]. Major efforts are in progress to develop a fungal based malaria mosquito control strategy, whilst the use of entomopathogenic fungi for the control of dengue vectors is also being investigated [6, 7].

Our group confirmed the potential of *Metarhizium anisopliae* for the control of the larval phase of *A. aegypti* [8], although the infective half-life of the conidia in water containing larvae was only 10 days. Other studies by our group have shown that the use of oil formulations can increase fungal persistence when used against adult *A. aegypti* [9]. *M. ansiopliae* was effective in reducing mosquito survival for up to 23 days when formulated in vegetable oil + isoparaffin and applied to black cloths fixed under furniture in test rooms simulating human dwellings. The survival rates were significantly lower than those observed for conidia formulated in Tween only or Tween + vegetable oil. Formulating conidia in vegetable oil + isoparaffin reduced the need for more frequent changes of black cloths in residences.

Oil formulations of *Metarhizium anisopliae* and *Beauveria bassiana* have also been tested against *Anopheles gambiae* larvae, facilitating application to water surfaces and improving persistence under field conditions [10]. When tested in Kenya, the percentage pupation of *An. gambiae* was significantly reduced by 39 – 50 % using ShellSol T-formulated *M. anisopliae* and *Beauveria bassiana* spores compared to results seen with unformulated spores.

The insecticidal activity of phytochemicals against mosquito larvae has been well documented (for review see: [11]), although no commercial products based on phytochemicals are currently being used in mosquito control programmes to our knowledge. One of the most extensively used “natural” plant derived insecticides is neem, extracted from the plant *Azadirachta indica* [12]. Commercially available neem based insecticides are used to control many crop pests and this is considered as a “green” approach to pest control, permitted in organic production systems.

Neem oil was shown to be toxic to *Anopheles stephensi*, *Culex quinquefasciatus* and *A. aegypti* larvae with median lethal concentrations (LC_50_) of 1.6, 1.8 and 1.7 ppm respectively. [13]. The neem oil formulation tested by Dua and co-workers [13] was also found to be effective in controlling mosquito larvae under natural field conditions.

Recently, entomopathogenic fungi have been formulated in neem oil and tested against larval and adult *An. gambiae* and adult *C. quinquefasciatus* [14, 15]. The results showed that the formulation of fungus + neem was more effective than neem alone for adults and larvae. The “formulation” of fungus in water was not as effective as fungus formulated in neem oil against adults, although larvae were not exposed to formulations of fungus without neem [15].

Our group has previously shown the beneficial effects of ultra-low concentrations of a neonicotinoid insecticide, imidacloprid, on the virulence of *M. anisopliae* when tested against adult *A. aegypti* [16]. Continuing our search for useful adjuvants, here we demonstrate for the first time that neem oil can also be used in combination with conidia of the fungus *M. anisopliae* for the control of *A. aegypti* larvae. This type of approach is important when aiming for rapid mortality of larvae knowing that fungal conidia have a limited half-life under field conditions.

## Methods

### Maintenance of insect colonies

*A. aegypti* (wild type strain) larvae were obtained from field collected mosquito eggs. Eggs were collected using “ovitraps” placed around the University campus. Only F1 larvae were used in all experiments. Larvae were maintained in plastic trays (80 larvae per 100 mL) and fed on freshly ground and autoclaved commercial mouse food (Nuvilab, São Paulo) (0.05 g per L). Only stage 2 and 3 larvae were used in experiments here.

### Fungal isolate and preparation of suspensions

The isolate of *M. anisopliae* used here was obtained from the collection at ESALQ (ESALQ818) in Piracicaba (São Paulo), which had been previously demonstrated to have high virulence against adult *A. aegypti* [7]. Fungi were cultured on Sabouraud Dextrose Agar (Dextrose 10 g; Peptone 2.5 g; Yeast Extract 2.5 g; Agar 20 g in 1 L H_2_0) at 27 °C for 15 days before being used in experiments. Fungal suspensions were initially prepared in Tween 80 (0.05 % in sterile distilled water) and conidial concentration determined using a Neubauer hemocytometer. Different conidial concentrations were prepared by serial dilution.

### Virulence test protocol

The tests were performed under laboratory conditions at 25 °C; 75 % RH 16: 8 L/D photoperiod. Conidia were formulated with 0.05 % Tween 80 (TW) or neem oil at the different concentrations used here. For tests, larvae were placed in 100 ml plastic cups with 50 ml tap water. For each test, three plastic cups with 10 larvae (stage 2 and 3) per cup were used with a total of 30 larvae per treatment. Each test was carried out three times.

### Preparation of neem oil

The commercially produced oil “Base Neem” from the company “Base Fertile Nim” (São Paulo, Brazil) with 0.12 % Azadirachtin, the main insecticidal component, was used in tests here. The oil was diluted with distilled water to the desired concentration.

### Effect of neem oil on condial germination and vegetative growth in vitro

For these assays, ESALQ 818 was used at a concentration of 1 × 10^8^ conidia ml^−1^ suspended in neem oil at three different concentrations: 1, 0.1 and 0.001 %. For the conidial germination assay, 15 μL aliquots of each formulation were inoculated onto SDA plates and maintained in an incubator (27 °C; 12 h L: 12 h D) for 12 h. The number of germinating conidia was evaluated using an inverted optical microscope (Biovera, Brazil). For each treatment, three Petri dishes were evaluated and three different regions of the plates were randomly observed. Conidia were considered to have germinated when the germ tube was equal or larger than the conidia. For the control treatment, conidia were suspended in 0.05 % Tween 80. Mean percentage germination rates between treatments were compared using one-way ANOVA and Duncan *post-hoc* test.

For the radial growth assay, the same procedure was used as above except that 50 μL of conidial suspensions in each formulation was inoculated in the centre of each SDA plate. The plates were maintained under the same conditions as above and radial growth measured on a daily basis for 7 days. Three plates were evaluated for each treatment. Mean growth rates between treatments were compared using one-way ANOVA and Duncan *post-hoc* test.

### Pathogenicity and virulence testing of the fungus *M. anisopliae* against *Aedes aegypti larvae*

To each beaker containing ten *A. aegypti* larvae, 1 mL of the fungal suspension was added. Five concentrations of the fungus were tested against larvae: 1 × 10^9^ conidia/ml, 1 × 10^8^ conidia mL^−1^, 1 × 10^7^ conidia mL^−1^, 1 × 10^6^ conidia mL^−1^ and 1 × 10^5^ conidia mL^−1^. The number of surviving larvae was recorded daily for 7 days. Dead larvae were removed from the plastic cups on a daily basis. The control treatment was carried out using Tween only.

### Toxicity of Neem oil against *Aedes aegypti*

The toxicity of neem oil against *A. aegypti* larvae was investigated. Concentrations of neem oil used here were 1, 0.1, 0.01, 0.001 and 0.0001 %. The number of surviving larvae was recorded daily for 7 days. Dead larvae were removed on a daily basis. The control treatment was carried out with distilled water. In this test, the concentration of neem oil to be combined with *M. anisopliae* was chosen based on the statistical analysis of the survival curves using ANOVA.

### Interaction of the fungus *Metarhizium anisopliae* with neem oil against *Aedes aegypti* larvae

The purpose of this test was to determine whether the combination of fungus + neem significantly reduced the survival rate of *A. aegypti* larvae when compared with tests performed only applying fungus or neem separately to water containing the larvae. The control treatment was carried out with Tween only. Two concentrations of fungus (10^7^ conidia mL^−1^ and 10^8^ conidia mL^−1^) were combined with the pre-determined “sub-lethal” concentration of neem oil (0.001 %) and tested against *A. aegypti* larvae. The number of surviving larvae was recorded daily for 7 days. Dead larvae were removed from the plastic cups on a daily basis. End point (7 days) statistical comparisons of survival rates were carried out using ANOVA.

All experiments were carried out three times with a minimum of 30 insects per treatment group or control group. The homogeneity of the replicate experiments was determined using the Log-rank test at the 95 % significance level and subsequently the results were pooled for survival curve comparisons between treatments (also using Log-rank).

## Results

### Effect of neem on condial germination and vegetative growth in vitro

Neem oil (1 to 0.001 %) had no effect on conidial germination or vegetative growth when tested *in vitro*. The results for percentage germination are shown in Additional file 1: Table S1 and for vegetative growth in Additional file 1: Figure S1.

### Toxicity of neem oil against *Aedes aegypti*

**Fig. 1:**
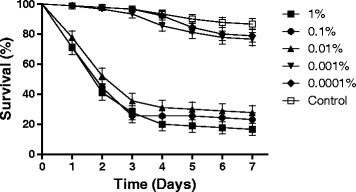
Daily survival curves of *Aedes aegypti* larvae exposed to different concentrations of neem oil. Note: Results are the means (± SE) of three experiments for each treatment with 30 insects used per treatment for each experiment

A 1 % concentration of neem oil resulted in the lowest larval survival rate (18 %) on the seventh day of evaluation, compared to the other concentrations tested here (Table 1). The survival of larvae exposed to 1 % neem oil was statistically equal to the survival rates of larvae exposed to 0.1 or 0.01 % neem (*p* > 0.01). The survival rates following exposure of larvae to these three concentrations of neem were significantly different to larval survival rates at all other concentrations [F _(5,17)_ = 22.218; *p* <0.01]. Figure 1 shows the daily survival rates of *A. aegypti* larvae exposed to five concentrations of neem oil. The survival curves were significantly different using the Log-Rank test (*χ*^2^ 199.4; df 5; *p* < 0.0001). The three highest concentrations grouped together, whilst the two lower concentrations were also similar, both causing low mortality rates. A concentration of 0.001 % neem resulted in larval survival rates (77 %) similar to those of the control group (86 %; *p* > 0.01). Therefore, this concentration was chosen for use in combination with *M. anisopliae* and further tested against *A. aegypti* larvae.

**Table 1:** Survival (%) ± Standard Deviation and Median Survival values for *Aedes aegypti* larvae exposed to five concentrations of neem oil

Neem Concentration	% Survival	S_50_
1 %	18.8 ± 11.8 b	2
0.1 %	25.5 ± 12.24 b	2
0.01 %	30 ± 10.0 b	3
0.001 %	77 ± 2.26 a	ND
0.0001 %	80 ± 2.50 a	ND
Control	86 ± 0.95 a	ND

The control treatment was distilled water only. Results followed by the same letter (comparing columns) indicate no significant differences when using Duncan’s post-hoc test (5 % probability). Values for S_50_ were calculated using Log-rank survival analysis. ND = Not determined

#### Pathogenicity and virulence of the fungus *M. anisopliae* against *Aedes aegypti* larvae

**Fig. 2:**
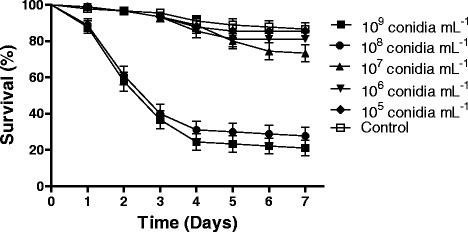
Daily survival curves of *Aedes aegypti* larvae exposed to different concentrations of *Metarhizium anisopliae* conidia. Note: Results are the means (± SE) of three experiments for each treatment with 30 insects used per treatment for each experiment

Survival rates of larvae exposed to different concentrations of conidia were significantly different from each other [F_6,20_ = 31.405 P < 0.01]. The tests carried out using *M. anisopliae* at a concentration of 1x10^9^ conidia ml^−1^ resulted in the lowest percentage survival of *A. aegypti* larvae (24 %) on the seventh day of evaluation (Table 2). However, the survival rate of larvae exposed to 1 × 10^9^ conidia ml^−1^ was statistically equal to the percentage survival of larvae treated with 1 × 10^8^ conidia ml^−1^ (30 %; *p* > 0.01). The larvae exposed to concentrations 1 × 10^9^ or 1 × 10^8^ conidia ml^−1^ both displayed S_50_ values of 3 days. Figure 2 shows the daily survival curves of larvae of *A. aegypti* exposed to five concentrations of the fungal isolate used here. The survival curves were significantly different using Log-Rank comparisons (*χ*^2^ 208.9; df 5; *p* < 0.0001). The survival curves for the two highest conidial concentrations were similar, whilst the three other fungal concentrations grouped together resulted in low levels of larval mortality.

**Table 2:** Survival (%) ± Standard Deviation and Median Survival values for *Aedes aegypti* larvae exposed to five concentrations of the fungus *Metarhizium anisopliae*

Treatments	% Survival	S_50_
1 × 10^9^ conidia mL^−1^	24.4 ± 10.5 b	3
1 × 10^8^ conidia mL^−1^	30.0 ± 9.86 b	3
1 × 10^7^ conidia mL^−1^	73.3 ± 2.5 a	ND
1 × 10^6^ conidia mL^−1^	81.1 ± 2.5 a	ND
1 × 10^5^ conidia mL^−1^	85.5 ± 1.77 a	ND
Control	88.8 ± 1.39 a	ND

The control treatment was Tween 80 (TW) 0.05 % in sterile distilled water. Results followed by the same letter (comparing % survival) indicate no significant differences when using Duncan’s post-hoc test (5 % probability). Values for S_50_ were calculated using Log-rank survival analysis. ND = Not determined

### Interaction of the fungus *Metarhizium anisopliae* and 0.001 % neem oil

For the first set of experiments with both agents, *M. anisopliae* at a concentration of 10^7^ conidia ml^−1^ was combined with a concentration of 0.001 % neem oil and tested against *Aedes aegypti* larvae.

**Fig. 3:**
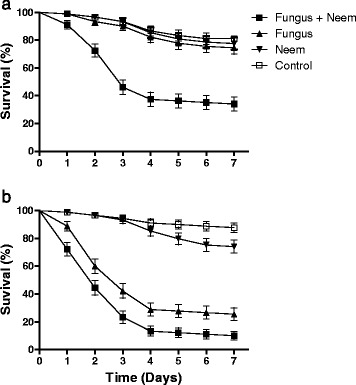
**a**. Daily survival curves of *Aedes aegypti* larvae exposed to *Metarhizium anisopliae* 10^7^ conidia mL^−1^, 0.001 % neem oil, and a combination of both agents (Fungus + Neem). **b**: Daily survival curves of *Aedes aegypti* larvae exposed to *Metarhizium anisopliae* 10^8^ conidia mL^−1^, 0.001 % neem oil, and a combination of both agents (Fungus + Neem). Note: Results are the means (± SE) of three experiments for each treatment with 30 insects used per treatment for each experiment. Control: Tween 80

The survival rate of larvae exposed to a combination of neem + fungus was 36 %. This was significantly different to the survival rates of larvae exposed to just the fungus (74 %) or the controls (81 %) [F _(3,11)_ = 18.3; *p* <0.01]. The survival rate of larvae treated with neem only (78 %) was statistically similar to that of the controls (Table 3; P <0.01). Figure 3a shows the daily survival rates of *A. aegypti* larvae from the different treatment groups. The survival curves were statistically different using Log-Rank comparisons (*χ*^2^ 76.67; df 3; *p* < 0.0001). The median survival (S_50_) was 3 days following exposure to fungus + neem.

**Table 3:** Interaction of Neem and *Metarhizium anisopliae* when using a conidial concentration of 10^7^ conidia mL^−1^

Treatments	% Survival	S_50_
F + N	36.6 ± 9.30 b	3
F (10^7^ conidia mL^−1^)	74.4 ± 2.36 a	ND
N (0.001 %)	78.8 ± 2.28 a	ND
Control	81.1 ± 1.90 a	ND

Survival (%) ± Standard Deviation and Median Survival for *Aedes aegypti* larvae exposed to a combination of either fungus + Neem (F + N); Fungus only (F) or Neem only (N)

The control treatment was Tween 80 (0.05 %) in sterile distilled water. Results followed by the same letter (comparing columns only) indicate no significant differences when using Duncan’s post-hoc test (5 % probability). Values for S_50_ were calculated using Log-rank survival analysis. ND = Not determined

A second set of experiments was carried out to test the combination of a higher concentration of fungus (10^8^ conidia ml^−1^) with neem (0.001 %). Table 4 shows the percentage of surviving larvae treated with this combination of neem and fungus (12 %) was significantly different from the percentage of surviving larvae treated with the fungus alone (28 %) or with neem alone (75 %; F _(3,11)_ = 156.04; *p* <0.01). Larval survival when exposed to neem alone was not significantly different (*p* <0.01) to the control survival values (90 %). Increasing the concentration of fungus slightly decreased the survival rate of larvae exposed to both fungus and neem when compared to the previous experiment, although it should be observed that the fungus alone at 10^8^ conidia ml^−1^ also resulted in low levels of survival. All survival curves were significantly different (*χ*^2^ 161.6; df 3; *p* < 0.0001). The S_50_ value for insects exposed to fungus alone was 3 days whilst a combination of fungus (10^8^ conidia ml^−1^) and neem resulted in a S_50_ value of 2 days. A two way analysis of variance was performed to compare the results for larval survival when using the two different concentrations of fungal inoculum. The survival rates when comparing the two conidial concentrations were shown to be significantly different (F_(1,4)_ = 39.2; *p* <0.01).

**Table 4:** Interaction of Neem and *Metarhizium anisopliae* when using a conidial concentration of 10^8^ conidia mL^−1^

Treatments	% Survival	S_50_
F + N	12.2 ± 11.5 d	2
F (10^8^ conidia mL^−1^)	28.8 ± 9.49 c	3
N (0.001 %)	75.5 ± 2.41 b	ND
Control	90 ± 1.11 a	ND

Survival (%) ± Standard Deviation and Median Survival for *Aedes aegypti* larvae exposed to a combination of either fungus + Neem (F + N); Fungus only (F) or Neem only (N)

The control treatment was Tween 80 (0.05 %) in sterile distilled water. Results followed by the same letter (comparing columns only) indicate no significant differences when using Duncan’s post-hoc test (5 % probability). Values for S_50_ were calculated using Log-rank survival analysis. ND = Not determined

## Discussion

The use of combinations of control techniques for the management of agricultural pests is well established and denominated as Integrated Pest Management (IPM). Although Integrated Vector Management (IVM) strategies are encouraged by the WHO, they are less often utilized in vector control programmes. This could be one of the reasons why insecticide resistance has quickly developed in vector populations. Research on the possible use of conventional insecticides together with biological control agents (BCAs), or the combination of BCAs with phytochemicals is a new and exciting field.

Here we investigated neem oil as a possible adjuvant for use with entomopathogenic fungi. It was possible to significantly increase the virulence of *Metarhizium anisopliae* against *A. aegypti* larvae by combining fungal conidia with relatively low concentrations of neem oil. Although neem can be used on its own to control mosquito larvae, this is unlikely to be economically viable for the large geographical areas that would need to be treated. Fungi are considered promising biological control agents for use against mosquito larvae, although any measures that could further reduce kill time in the field are of great interest. We have previously shown that an isolate of *M. anisopliae* (CG 144) was capable of causing a 90 % larval kill rate under laboratory conditions over a 7 day period [8]. Another isolate of *M. anisopliae* (ESALQ 818) was also highly virulent to *Aedes* larvae, with a S_50_ of 2 days. This isolate was shown to have a half-life of 10 days in tests carried out under laboratory conditions.

A recent study has shown that an isolate of *M. anisopliae* was highly efficient at killing *A. aegypti* larvae, with 60–90 % mortality observed 72–96 h post-inoculation, however, larval death was not the result of a normal infection process [17]. Butt and co-workers [17] presented evidence that insect mortality appears to be linked to autolysis through caspase activity regulated by Hsp70, and this response can be modified by protease inhibitors. An abnormal insect-fungus interaction was attributed to the fact that *M. anisopliae* is a terrestrial fungus and is therefore not adapted to an aquatic environment.

The potential use of phytochemicals for the control of insect vectors has been the subject of many studies (for review see: [11]). Amongst these bioactive compounds, neem oil is one of the few to have been commercially successful in agricultural regimens. Neem tree extracts have also been tested for toxicity against mosquito larvae and have been shown to kill *Aedes*, *Culex* and *Anopheles* [13, 15, 18].

Among the compounds found in neem seed, azadirachtin is the main biologically active component. The presence of azadirachtin in neem seeds has been highly correlated with bioactivity against insects [19]. At a physiological level, it has been shown that azadirachtin blocks the synthesis and release of developmental hormones such as the ecdysteroids, leading to incomplete moulting of immature insects. Its action as a growth regulator weakens the defense system of the larval cuticle, facilitating the penetration of pathogenic organisms [20]. In adult females, a similar mechanism of action leads to sterility, besides presenting anti-feedant properties [12, 21, 22].

The lethal effects of neem based products were evaluated against *A. aegypti* larvae and have been shown to affect development of immature stages of this insect, resulting in significant reduction in adult emergence [13, 23]. In the case of the malaria vectors, *An. stephensi* and *An. gambiae*, the administration of pre-determined doses of neem led to inhibition of feeding and reduction in oviposition as well as significant reduction in the formation of pupae and reduced adult emergence [23–25].

The toxicity curves of the commercial neem product used here were grouped together into three concentrations with high toxicity (0.1 % – 1 %) and two concentrations, which displayed low toxicity (0.001 and 0.0001 %). The three highest concentrations resulted in between 18 and 30 % survival, whilst the two lower concentrations resulted in 77–80 % survival. The highest concentration of neem tested here (1 %) resulted in a 50 % reduction in survival rates over 2 days. Other researchers have shown that neem oil caused 95 % mortality when used at a concentration of 5 % against *An. stephensi* larvae [18]. These workers also described the effect of neem oil on pupal development, with 5 % neem totally inhibiting the formation of pupae, whilst 3 % neem oil caused an 11 % reduction. Neem seed kernel extracts were also shown to prolong the larval development phase in *A. aegypti* when 1^st^ instar larvae were continuously exposed to the extracts [26].

The use of a combination of neem oil and *M. anisopliae* were tested against adult *An. gambiae* and *C. quinquefasciatus* by spraying this formulation onto the netting of cages into which adult mosquitoes were subsequently released [14]. By day 4 of the experiment the fungus + neem formulation resulted in a 4 % survival rate for *An. gambiae* and 12 % for *Culex*, which was highly effective when compared to neem alone (82 and 89 % survival respectively) and fungus in water (58 and 70 % survival respectively). In the study of the effects of neem oil formulations of fungal conidia against larvae of *An. gambiae*, the same authors recorded modifications in adult emergence following treatments over an 8 day period [15]. Neem formulated fungal conidia reduced adult emergence to around 2 % whilst neem alone reduced adult emergence to around 28–30 %. However, without testing the fungus only, the actual contribution of the entomopathogen in reducing adult emergence remains unknown.

Before selecting a suitable adjuvant for application with entomopathogenic fungi, it is necessary to test for possible toxicity to the biological control agent. Although many chemical insecticides are compatible with entomopathogenic fungi, some have been shown to have deleterious effects on these organisms. One of the new generations of insecticides, the neonicotinoid imidacloprid, was shown to have no effect on the germination of *B. bassiana* conidia [27]. However, some studies have shown that neem oil is not compatible with *B. bassiana*, inhibiting conidia vegetative growth and decreasing conidial production and viability [28]. Neem oil was moderately toxic to *B. bassiana* when used at 0.5 % and highly toxic at 1.5 %. However, neem seed and leaf extracts were compatible with this fungus. Another study showed that using neem oil at less than 1 % caused no deleterious effects on the fungus *Metarhizium acridum* and the combination of neem oil with this fungus accelerated locust mortality and increased efficiency of the biological control agent [29].

Here we show that when using a combination of 1 × 10^7^ conidia mL^−1^ + neem at a final concentration of 0.001 %, the survival rates were 36 % as compared to applications of the fungus alone which resulted in 74 % survival. The results for larvae exposed to this concentration of neem alone resulted in 78 % survival. In the experiments using a higher concentration of fungal conidia (1 × 10^7^ conidia mL^−1^), the survival curves were different, with this combination of fungus + neem resulting in the lowest survival rates seen here (12 %). At the higher fungal concentration, survival rates were also significantly reduced (28 %) when compared to those using 1 × 10^7^ conidia mL^−1^ alone. As was observed by other workers, combining two control agents requires extensive research to obtain the desired effect. Our aim here was to verify the concentration of neem oil required to increase larval mortality when combined with the fungus without having a negative effect on the fungus itself. As we show here, all neem oil concentrations tested had no effect on the fungus, however, from an economic view point, using low concentrations of neem is advantageous. The results also show that using higher concentrations of fungal conidia alone significantly reduce survival rates. Even so, combining higher concentrations of conidia with low concentrations of neem also had beneficial effects, low total survival and low S_50_ values. It should be remembered that these assays were carried out under laboratory conditions and further tests under field conditions should be performed.

## Conclusions

The current approach using a combination of a phytochemical and entomopathogenic fungus has multiple benefits, apart from increasing total mortality and reducing time to kill, neem could also increase fungal persistence and reduce the chances of resistance development which has been shown to occur when using mixtures of control agents [30].

## Additional file

Additional file 1:
**Table S1.** Percentage conidial germination at 16h post-inoculation on culture media. **Figure S1.** Radial growth rates of Metarhizium anisopliae when formulated in different concentrations of neem oil over a 7 day period. (DOC 63 kb)
