# Supplementary material for: Neem oil increases the efficiency of the entomopathogenic fungus Metarhizium anisopliae for the control of Aedes aegypti (Diptera: Culicidae) larvae
Source: Parasit Vectors. 2015 Dec 30;8:669. doi: 10.1186/s13071-015-1280-9 (PMC4696216; doi:10.1186/s13071-015-1280-9)
Supplement: Additional file 1: — Table S1. Percentage conidial germination at 16h post-inoculation on culture media. Figure S1. Radial growth rates of Metarhizium anisopliae when formulated in different concentrations of neem oil over a 7 day period. (DOC 63 kb) [file 13071_2015_1280_MOESM1_ESM.doc]

**Table S1**: Percentage conidial germination at 16h post-inoculation on culture media.

|  | **Percentage Germination** |
| --- | --- |
| **F** | 76.2 a |
| **F+N 1%** | 72.8 a |
| **F+N 0.1%** | 73.4 a |
| **F+N 0.001%** | 74.3 a |

There was no significant difference between treatments (F_3,11_ = 0.933 ; *p*>0.01)

F: fungal conidia; N: Neem (% concentration)

**Figure S1**: Radial growth rates of *Metarhizium anisopliae* when formulated in different concentrations of neem oil over a 7 day period.

There was no significant difference between growth rates when compared on day 7 (F_3,11_ = 0.833; *p*>0.01).
